# Report of the Committee on Dental Nomenclature

**Published:** 1893-11

**Authors:** G. V. Black


### REPORT OF THE COMMITTEE ON DENTAL NOMENCLATURE.[1]

BY DR. G. V. BLACK.

THE task assigned to this committee, to "present a plan by which a universal system of nomenclature may be adopted by the Congress that would be acceptable to the profession of the entire world," is an exceedingly difficult one.

Accepting Webster's definition of the term, our nomenclature consists of the names which we apply to *things* or *ideas*, with their accompanying adjectives or words designating relation or location. A specified form of nomenclature requires an adjustment of phraseology to suit the forms of names used, thus giving an impress to the entire style, and in some degree to the plan of writing. The plans of expression of thought must be adjusted to the nomenclature employed, or the nomenclature must be adjusted to the plans of the expression of thought. It generally happens that in the early growth of any science, the nomenclature is in a great degree adjusted to the modes of expression of the individual writer. It is in this condition that we find dental nomenclature to-day, and it is this tendency to individual characters in writers, often good in itself, that forms one of the greatest difficulties in the way of the adoption of a definite scheme of nomenclature.

The nomenclature of any science or art is a growth, and it has been necessary to trace the steps of the growth of dental science

---

[1] Read before the World's Columbian Dental Congress, Chicago, 1893.

in order to understand the formation of its nomenclature, and gain a clear conception of its future tendencies. We cannot sit down and construct a nomenclature for dentists any more than we can arbitrarily prescribe the words to be used by any other class of people. Forms of speech are not readily adopted at will, but come to classes of people by processes of growth and education. Hence for a single person, or a committee selected from a class of persons, to arbitrarily prescribe the forms of speech for the class, taking their own individuality as their guide, would be wrong in theory and fact, and would only invite failure. The adoption of a definite scheme of nomenclature should be brought about slowly, and after full discussion of the scheme developed by the custom of the best writers in the particular field of science. This scheme may then be improved in the detail of its workings from point to point until finally the individual forms of the words used may be prescribed as is now done in botany, zoology, and other natural sciences.

In dentistry we may be said to have no existing rules except such as may have been developed by the customs of writers; rules not recognized by the writers themselves, unless, indeed, they have undertaken some extended study of forms of nomenclature in general, and the systematization of their own. The effort of systematization seems to have been rare among dentists, and they seem not to have given much study to the general subject of nomenclature. The studies that your reporter has been able to find generally have reference to the adoption of some individual words, or groups of words in a given language. Nowhere in dental literature do we find studies which seem to have direct reference to the systematization of dental nomenclature as a whole.

Your reporter has found no recognized rules of dental nomenclature that will serve as a basis upon which to proceed to the improvement of existing forms. We are therefore without a basis of action until such a scheme is made known and recognized in such a way as to form the basis of discussion.

The necessity for a definite system of nomenclature is becoming more and more apparent to many of the more thoughtful men. Dental literature is being rapidly built up, and within a few more years the more mature thought of the profession will be moulding into a more permanent form, or a form from which changes will be much less rapid than they have been in the past. Many have a desire to see this literature take on a form of nomenclature that will be regular, concise, and homogeneous, uniting all its parts in such a way that the acquisition of its facts will be easy to the student, and so that writers and speakers will be able to present their thought with accuracy and be readily understood by readers and hearers.

A chief difficulty in the outstart of a work of this kind is to fix a standard of beginning. After such a standard has been established and rules have been formed, a revision is much less difficult. Fixing the standard of beginning, or the scheme of nomenclature for dentistry, involves the fixing of a basis list of names to be used in the future, the form and source of which will become the guide for the future formation of names.

The formation of both the scheme and the detail will be a difficult matter, and will require much time for several reasons. The custom followed by the older sciences in the establishment of rules of nomenclature requires that all original workers in the particular field of science be consulted, or at least that each shall have the opportunity to express his views. In our own specialty, your reporter has gathered the names of all who have written journal articles and books in the English, French, German, and Italian languages within the last two and a half years, and finds the number of persons to be two thousand eight hundred and sixty-five. The number of journal articles presented in this time, exclusive of editorials, is six thousand three hundred and fourteen. They are distributed as follows:

| | Persons. | Journal Articles. | Books. |
|---|---|---|---|
| English language, America . . . . . . . . | 1152 | 2623 | 51 |
| " " England . . . . . . . . | 519 | 1116 | 11 |
| " " Canada . . . . . . . . | 58 | 72 | 0 |
| German language . . . . . . . . . . . . | 610 | 1372 | 58 |
| French language. . . . . . . . . . . . . | 479 | 1029 | 39 |
| Italian language . . . . . . . . . . . . | 47 | 102 | 5 |
| Totals . . . . . . . . . . . . . . | 2865 | 6314 | 164 |

(The names obtained from other countries probably do not properly represent them, and their numbers are not given. It seems that all who have written books appear also in journal articles.)

To undertake to harmonize the views of this large body of workers is a great task, and steps should be taken to bring the matter before them for general discussion, with the view of finally adopting some fixed scheme and rules for the regulation of the selection of technical terms. What is here called the scheme involves the selection of the source of names and their form. In the nomenclature of botany and zoology the scheme requires that names shall have the Latin form,—words from the Latin language, or latinized vernacular words. As time has passed and the use of the Latin tongue has diminished the use of the Latin, case endings have been abridged more and more until now, in the general use of the words, they are practically limited to a single form of termination.

[Dr. Black then, after detailing the history of dental nomenclature as at present existing, suggested the following scheme as a basis for work.]

The scheme, as has been said, involves the fixing of a starting-point, or the fixing of the forms and the sources of the words to be employed in dental nomenclature. This is embodied in the following:

1. The plan of nomenclature shall be the same in the several languages.

2. Use words derived from the Latin or Greek whenever such words are available, making use of the root and giving it such terminations as may be suited to the language in which it is employed.

*Note.*—When a word in the Latin or Greek form has come into favorable use in any language, there will be no necessity for changing it to the vernacular form.

3. When, for any purpose, a word from the Latin or Greek is not available, agree upon a word from another language and use in the same way.

4. When it is impracticable to use the same word in the several languages, select a word from each vernacular language. These should be as nearly exactly translatable as possible.

5. Adopt such general and specific rules employed in other sciences as may be adapted to dental nomenclature.

[Proceeding with the development of his subject, Dr. Black offered the following rules as suggestions:]

Rule 1.—Carious cavities shall be designated by the names of the surfaces of the teeth in which they occur.

*Note.*—These names are: *Labial*, for the incisors and cuspids; *Buccal*. for the bicuspids and molars; *Lingual*, for all of the teeth, discarding the word *palatal; Mesial* and *distal* for the proximate surfaces; *Occlusal* for the bicuspids and molars, and *incisal*, for the cutting-edges of the incisors and cuspids. (Dr. Andrieu has used the term *cuspidale—i.e.*, *disto-cuspidale*—for cavities involving the edge, or cusp, of the cuspids; and also the term *buccal* for all of the teeth, instead of using *labial* for the six anterior teeth.)

Rule 2.—When two surfaces are involved, the names of the surfaces are rendered in compound words, giving the preference, first, to mesial and distal as the prefixes, and second, to occlusal. In doing this drop the final *al* and add *o*; thus, *disto-lingual*, *mesio-occlusal*, *occluso-buccal*, *disto-incisal*, etc.

Rule 3.—When three or more surfaces are involved, the names of the individual surfaces are rendered in a compound word; thus, *Mesio-occlusal-distal cavity*. (See exhibit of cavity names.)

Rule 4.—Cavities in the angles of the teeth are designated by the anatomical names of the angles in which they occur; thus, *disto-buccal angle cavity*; *mesio-lingual angle cavity*, etc.

Rule 5.—As a class name mesial and distal cavities are called *proximate cavities*; thus, *proximate cavities* in the *molars*.

Rule 6.—In describing the superficial extent of cavities, use no words indicating direction or position upon the teeth, except the names of the surfaces, the adverbs derived from those names, the divisions of the surfaces into thirds, or the anatomical names of parts or surface markings of the teeth; as the lobes, cusps, angles, grooves, pits, etc.

The report, which was an exhaustive presentation of the subject, entering into a considerable exposition of details, was accompanied by an "Exhibit of the nomenclature of dental anatomy," as compiled from a number of authors in French, German, and American writers, showing the gradual development of terms. It closed with the following recommendation:

These considerations lead your reporter to recommend that a commission be formed to take up this work and complete a basis of beginning, with such rules as their information may suggest as most practicable, and bring this before the whole profession for discussion of all of its features; making the best possible provision for its discussion in all dental journals, societies, and associations. And that they, from the information that they may in this way obtain of the wishes of the profession, revise both the basis and the rules, and finally present their report for adoption by such body as may be appointed to receive it.

